# Ultrafast Imaging using Spectral Resonance Modulation

**DOI:** 10.1038/srep25240

**Published:** 2016-04-28

**Authors:** Eric Huang, Qian Ma, Zhaowei Liu

**Affiliations:** 1 Department of Physics, University of California, San Diego, 9500 Gilman Drive, La Jolla, California 92093-0407, USA; 2 Department of Electrical and Computer Engineering, University of California, San Diego, 9500 Gilman Drive, La Jolla, California 92093-0407, USA

## Abstract

CCD cameras are ubiquitous in research labs, industry, and hospitals for a huge variety of applications, but there are many dynamic processes in nature that unfold too quickly to be captured. Although tradeoffs can be made between exposure time, sensitivity, and area of interest, ultimately the speed limit of a CCD camera is constrained by the electronic readout rate of the sensors. One potential way to improve the imaging speed is with compressive sensing (CS), a technique that allows for a reduction in the number of measurements needed to record an image. However, most CS imaging methods require spatial light modulators (SLMs), which are subject to mechanical speed limitations. Here, we demonstrate an etalon array based SLM without any moving elements that is unconstrained by either mechanical or electronic speed limitations. This novel spectral resonance modulator (SRM) shows great potential in an ultrafast compressive single pixel camera.

The need for high-speed imaging has led to a variety of strategies for capturing images at a very high frame rate. By storing the recorded measurements locally on-chip, ultrafast CCD cameras have been fabricated that can reach frame rates of up to 1MHz^1^. However, they are limited by the local storage space available on the chip, so currently this kind of cameras can typically record only 256 frames in one continuous run. Techniques based on the STREAK camera^2^,^3^ have managed to achieve high speed imaging by using a photocathode and electrode deflector to map points in time to locations on a phosphor screen, but again this imaging rate is only possible for a short period of time. Both of these imaging techniques are single-shot measurements that are suitable for events that can be predicted or triggered, but cannot be used for arbitrary or transient events. For periodic or repeatable phenomena, it is possible to use pump-probe techniques^4^,^5^ to simulate high imaging speeds, but they still cannot capture transient or non-periodic phenomena. A relatively new development is the STEAM camera^6^,^7^,^8^, which uses chromatic dispersion to encode the spatial information of an image into a serial time-based measurement, and has demonstrated real-time imaging frame rates of 6.1 MHz. Most these techniques, though, form a 2D image by recording data from each pixel in a large format sensor array, one measurement for each pixel. There is, however, a way to even further increase the imaging speed by overcoming the low efficiency intrinsic to the pixel-by-pixel data collection method.

Compressive sensing^9^,^10^ is a method for recovering a signal from a measurement made with a known sensing matrix. The term ‘compressive’ is used to denote the fact that an object with *m* values can be reconstructed with *n* measurements where *n* < *m,* similar to how it is possible to compress a digital image file to a much smaller file size using a compression algorithm such as JPEG^11^. It is a powerful tool, as the same data can be recovered by many fewer measurements, and thus has been used in a wide variety of applications^12^,^13^,^14^. The most common CS imaging method is the *single pixel camera*^13^,^15^,^16^,^17^ (SPC), in which the unknown image is sequentially sampled by known patterns using a spatial light modulator (SLM), and the resulting total intensity is measured using a photodetector. The *m* pixels of a 2D image are therefore encoded into the *n* measurements measured by the photodetector. In experiment, under-sampling ratios (*n/m)* of 10 percent or less are commonly achieved. Since the SPC is a serial imaging method, the under-sampling ratio directly results in shorter image acquisition times. Unfortunately, in spite of the improved image collection efficiency provided by CS, current SPCs typically are fairly slow due to the speed limit of the SLMs. The most widely used SLMs are based on liquid crystal modulators or digital multimirror devices (DMDs), which have maximum switching rates on the order of 10 kHz (~10^4^) due to mechanical limitations. In comparison, the electronic bandwidth of many sensitive photodetectors are around the GHz regime (~10^9^). As a result, while the SPC drastically improves the data acquisition efficiency, in practice the achievable imaging speed is bottlenecked by the available high speed SLMs. Here we would like to introduce a new method for spatial light modulation we refer to as a spectral resonance modulator (SRM) that bypasses the need for mechanical or electrical actuation, which allows for extremely high modulation speeds.

The basic building-block of the SRM presented here is the Fabry-Perot (FP) cavity (Fig. 1), also known as an etalon. The FP cavity consists of two parallel surfaces with a reflectivity of *R* and a separation of distance *d* between them by a material with an index of refraction *n*. The etalon transmittance (examples shown in Fig. 1a) is given by:


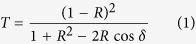


where δ = (2π/λ)·2*d*·*n*cos*θ*, and *θ* is the angle of light travels through the etalon. This wavelength-dependent transmission signature is highly sensitive to the optical thickness *d* (see Fig. 1(a) bottom panel), and can be used to encode transmitted light^18^. If, instead of a single FP cavity, we have an array of cavities with different thicknesses (Fig. 1c), we will now create a wavelength-dependent transmission array, where the output pattern is controlled by the wavelength of the illuminated light (Fig. 1d–f). Although arrays of tunable FP cavities have been used for things such as reflective displays^19^, we will use their characteristic transmission spectra instead to distinctly encode each FP cavity as a pixel in our final image. As our method does not require tuning of the cavity length, we can use a robust array of static cavities.

Since this FP cavity array is a completely passive device, it is not bound by either mechanical or electronic speed limitations. Instead, the maximum modulation rate is only limited by the response time *t*_*c*_ of the optical resonance:


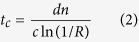


where *c* is the speed of light in vacuum. We will use this kind of SRM as the basis of our ultra-fast SLM.

## Results

**Figure 1:**
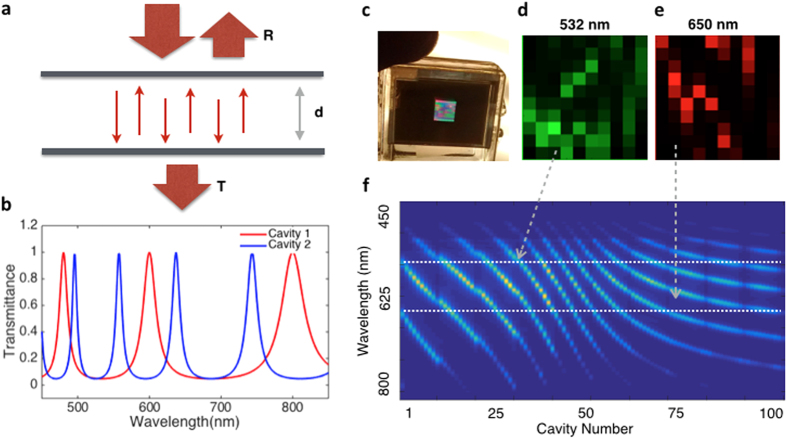
Principles of the Spectral Resonance Modulator (SRM), i.e. Etalon Array Modulator. (**a**) A Fabry-Perot cavity, i.e. etalon, consists of two semi-reflective surfaces separated by a distance d, and has a characteristic spectral reflectance R and transmittance T that are wavelength dependent. (**b**) The transmission spectrum for two etalons of different thicknesses will be unique. (**c**) A photograph of our fabricated 10 × 10 etalon array. (**d**,**e**) The transmission pattern from the etalon array at 532 nm and 650 nm. (**f**) The measured transmission spectrum for all 100 different etalons when illuminated by our laser, with the pictured transmission images (in **d**,**e)** marked with the dotted lines.

Our test SRM consisted of a 10 × 10 array of FP cavities (Fig. 1c–e) fabricated using grayscale electron beam lithography combined with thin film depositions (see Methods). The cavity length varies from 1.5 to 3.5 microns in 20 nm increments, and the mirror reflectivity R is about 0.8. The modulation time is set by the thickest (and therefore slowest) cavity response time in the array, and is around 67 femtoseconds for the 3 micron cavity. This translates into a time-bandwidth cut-off of about 15 THz. When the etalon array is illuminated by a white light source, the transmitted intensity shows different patterns at different wavelengths (see Fig. 1c–e). By adding an aperture to the sample plane and translating it across the image of each FP cavity, we were able to directly measure the transmission signature of all the individual FP cavities in both time and spectrum with the avalanche photodiode (APD) and the spectrometer (see Fig. 1f). The image of the FP cavities are much larger than the diffraction limit (~50 microns for each cavity), and so issues due to blurring or defocusing are minimized in the calibration.

However, an etalon array SRM alone is not equivalent to a SLM. A SLM is able to form different spatial patterns at different times, but a SRM only creates patterns with different wavelengths. A dispersive optical element is also needed to separate those wavelengths in time so that the etalon array SRM function as an SLM.

**Figure 2:**
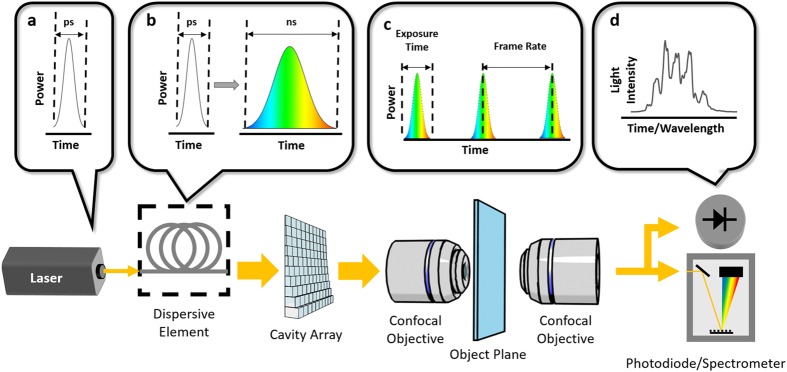
Schematics of the Experimental Set-up. A broadband laser pulse (**a**) is emitted, and time-stretched with a grating and a dispersive fiber so that the ps pulse becomes a ns-scale frequency sweep (**b**) (see Chromo-modal dispersion in Methods). The resulting light is sent through the etalon array, and the resulting light is now patterned in space, wavelength, and time. For our time-based measurements, the shutter speed will be related to the total pulse stretching time, and the frame rate will be related to the laser pulse repetition rate (**c**). After being projected onto an object and then collected with a pair of confocal objectives, the resulting light is measured either with a photodiode or a spectrometer (**d**). This measurement is the transmission of the object encoded with the projection pattern, and the data is then sent to a compressive-sensing algorithm to recover the image of the object.

Our proposed imaging method using the etalon array SRM is shown in Fig. 2. A broadband supercontinuum pulse laser (Fig. 2a) is transmitted through a device for dispersive pulse stretching (see Fig. 2b and ‘Chromo-modal dispersion’ in Methods), so that different color of light comes out at different time, and then used to illuminate the etalon array SRM, which creates the wavelength-dependent and time-dependent patterns (see examples in Fig. 1d,e). These patterns then illuminate an imaging target, and the transmitted light becomes encoded with the information from the target. Afterwards, the light is collected and directed to either an optical spectrometer, which records the transmitted spectrum, or an ultra-fast photodetector, which then records the resulting temporal pulse shape (Fig. 2d). This data is then sent to a compressive sensing algorithm, which recovers the image of the target.

**Figure 3:**
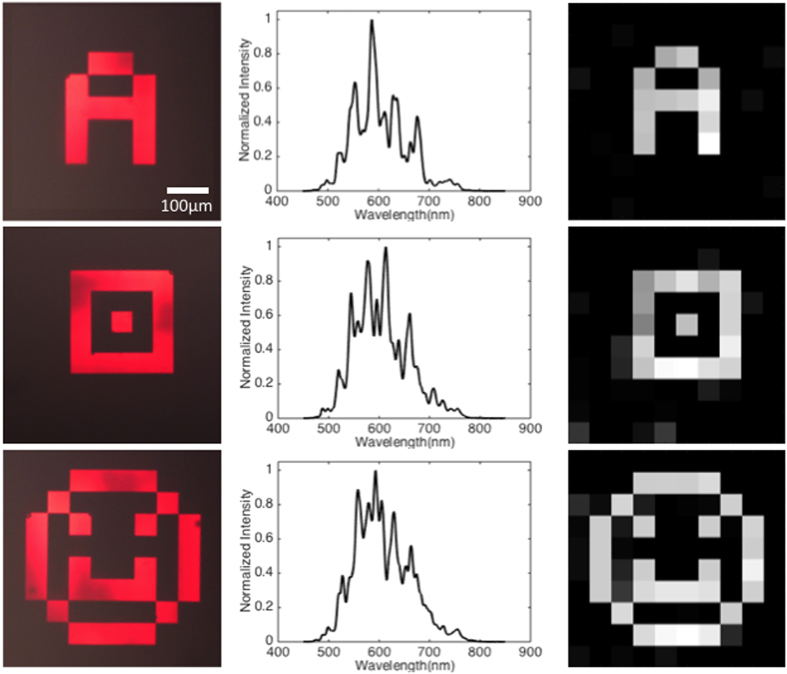
Reconstruction Results from Spectral Measurements. (**a**) Transmission images of the chrome objects captured by a CMOS camera. (**b**) The measured transmission spectra from the three different targets by using our proposed setup. (**c**) The reconstructed images from the measured spectra.

The experimental spectral imaging performance of the SRM based imaging system is shown in Fig. 3. After placing a variety of imaging targets in the sample plane (Fig. 3a), we capture the resulting transmission spectra (Fig. 3b), which contains the information from the image encoded into the FP cavity responses. Afterwards, we used a minimization algorithm (see methods) to reconstruct the images of the targets (Fig. 3c).

**Figure 4:**
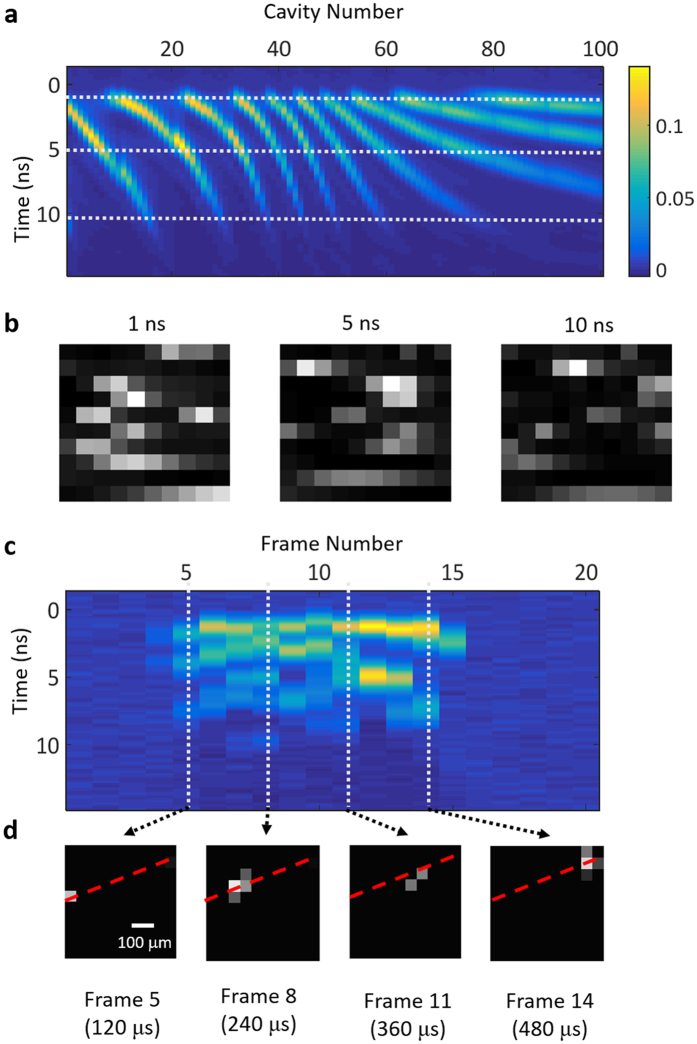
High-speed Image Reconstruction Results. (**a**) Temporal APD response (Volts) for the 100 individual etalons when illuminated by our stretched laser pulses. Cavity responses at 1, 5, and 10 ns are marked by dotted white lines. (**b**) The real-time detected light patterns of the etalon array at 1, 5, and 10 ns. (**c**) 20 real-time APD measurements (Volts) for a pinhole traversing the imaging area. Each frame was taken with a shutter speed of 12 ns and a frame rate of 25 KHz. The object enters the field of view in frame 4, and exits in frame 16. Selected frames are marked with a dotted line. (**d**) Reconstructed images of the selected frames. By using the known cavity response from (**a**) the position of the pinhole can be recovered. The dashed red line shows the trajectory of the pinhole as it moves across the field of view.

After showing successful image reconstruction using spectral encoding, we wished to also demonstrate imaging in the time domain as well. As previously estimated, the optical bandwidth limit of the SRM is around 15 THz, which is much faster than the rest of the whole system. In practice, the imaging speed limitation of our SRM based camera is mainly determined by the electronic bandwidth of the detection system and the amount of dispersive time-stretching. In our prototype, the bandwidth of our avalanche photodiode extends to 1 GHz. Our method of chromatic pulse stretching (see Methods) can currently stretch the pulse envelope to a maximum of 12 ns. As with the FP cavity spectrum, the total pulse stretching time will set a limit on the sparsity of the object to be imaged. However, this type of limitation is well suited for high-speed tracking or localization applications where the imaged object is very simple. To test the real-time measurements of our system, we put a spinning disk with a pinhole in our sample plane, and measured the response of our avalanche photodiode (APD) as the pinhole traversed the imaging area (Fig. 4). This resulted in a train of single-shot temporal measurements of the light intensity, where each measurement was the response of the structured pulse of light encoded by the transmission of the pinhole. Afterwards, by using the previously measured temporal responses of all of the FP cavities, we were able to reconstruct the path of the pinhole as it moved across the field of view.

## Discussion

Our proof of concept experiment, however, is still far from its theoretical constraints. In practice, our frame rate is set by the repetition rate of our laser, which for this experiment was 25 kHz. However, the highest theoretical imaging speed is set by the shutter speed, which is determined by the maximum achievable dispersive pulse stretching. In our experiment, we were able to achieve maximum pulse times of 12 ns (see methods). The number of achievable measurements can then be estimated by the pulse time multiplied by the minimum feature that the dispersion/sensing system can detect.

To estimate the imaging performance, we consider the following: number of measurements (m), total number of pixels (n), sparsity of the object (S), and the coherence between the sensing basis and the object (μ)^10^. Although there is no deterministic way to determine what a sufficient parameter is, in general good reconstruction will be more likely to succeed when equation 3 is true:


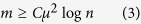


where C is an unknown constant. Currently, due to fabrication constraints, our prototype FP cavity array is 10 × 10 (n = 100), but there are no theoretical barriers to making a larger array, though it comes at a greater cost of either an increase in m or a decrease in S and μ. S and μ are both dependent on the object to be imaged, but μ in general is minimized when the sensing pattern is completely random. Since the wavelength transmission of a FP cavity is ordered and not random, in some sense the FP cavity is not ideal. However, this can be minimized by arranging the FP cavities randomly in space, although we did not do this in our prototype array for ease of fabrication. In general, there are many areas of possible improvement, which is aided by the fact a large number of parameters are easily tunable, allowing the imaging setup to be improved and targeted for specific imaging applications.

In conclusion, we have shown an extremely fast SLM by combining an engineered SRM, i.e. array of etalons, and a dispersive pulse stretcher. We also demonstrated the capability to use the SRM to achieve high speed compressive imaging. The prototype is currently capable of a single-shot image in a 12 ns single exposure. Beyond this prototype, we envision the principle of using SRM and CS for ultrafast imaging to be broadly suitable for a variety of different applications, particularly particle localization, which due to the inherent sparsity of the image, can allow for extremely high frame rates to allow for the tracking of very fast dynamic movements.

## Methods

### SRM Fabrication

The FP cavity array SRM was fabricated in multiple steps. First, we used sputtering deposition (Denton Discovery 18) to put a 30 nm silver layer on a glass microscope slide, and then deposited 70 nm of SiO_2_ using PECVD (Oxford Plasmalab PECVD) on top. Afterwards, we spin-coated a 2.8 μm thick layer of PMMA (MicroChem 950PMMA A9). Then, we used electron-beam lithography (Raith GmbH Raith50) to write a grid of 10 by 10 squares with lateral dimensions of 250 μm by 250 μm with doses varying from 1.4 μC/cm^2^ to 80 μC/cm^2^ at an energy of 10 keV, and then developed the sample for 5 minutes in methyl isobutyl ketone (MIBK), followed by a 30 second rinse with isopropanol, which etched the height pattern into the PMMA between 0–2 μm, resulting in the final optical thicknesses from 1.5 to 3.5 microns. After drying under N_2_, we then added the final 30 nm silver layer using sputter deposition, as well as a thin (~30 nm) protective SiO_2_ layer on top.

### Optical Measurements

Our pulsed laser is an NKT photonics SuperK COMPACT super-continuum laser, with an NKT VARIA tunable broadband filter. For the spectral measurements, we used a bandwidth range from 450–840 nm, and for the temporal measurements, we used a limited bandwidth from 620–840 nm. Our measurements were taken using either an optical spectrometer in the frequency domain (Andor Shamrock 303i with an Idus 420 CCD) or an avalanche photodiode (MenloSystems APD210) connected to an Agilent MSO9254A for real-time measurement.

### Chromo-Modal Dispersion

In order to measure the modulated output of the SRM in the time domain, a frequency-to-time conversion must be done using dispersive time stretching. Our method of doing so is referred to as chromo-modal dispersion (CMD)^20^. Our chromo-modal dispersion device uses a pair of blazed gratings (Thorabs, 300 grooves/mm separated by 10 cm) to separate the wavelengths of the input laser in space, and then re-collimate them. An objective lens (20x Olympus Plan Achromat, 0.39 NA) is then used to couple the light into a 100 m multi-mode optical fiber (Thorlabs BFL48-400). Due to the spatial separation of the wavelengths, this results in a wavelength-dependent angular occupation of the propagating fiber modes, with a corresponding wavelength-dependent group velocity. As a result, by the time the laser pulse propagates to the end of the fiber, the total pulse is stretched by about 12 ns for a bandwidth from 620–840 nm. This results in a total average dispersion of 545 ps/nm*km. However, scattering from within the fiber itself limits the quality of the pulse stretching, and the individual wavelengths of the incident light can be measured to have a pulse width of between 0.5 to 1 ns. At the end of the fiber, a mode scrambler was clamped to the fiber jacket, which forces the fiber through a series of tight bends that homogenizes the different modes and results in a uniform output from the fiber. More information about the performance of the CMD fiber can be found in supplemental information.

### Spectral and Temporal Calibration

In order to successfully reconstruct an image, the response from the Fabry-Perot cavities need to be measured very accurately. This was done by overlaying a square aperture at the sample plane of each individual FP cavity, then averaging the resulting waveform many times to recover the noise-free temporal or spectral response of the cavity. When taking the static imaging measurements, we then apply a patterned chromium mask to the sample plane, and take a single-shot measurement with the spectrometer. To take the real-time tracking measurement, we used a pinhole aperture connected to a spinning disk in the sample plane, and then recorded the captured waveform from the APD.

### CS Reconstruction Algorithm

The algorithm used for the compressive sensing reconstruction is described by Kim *et al*.^21^, which uses the L1-norm as a basis for minimization. The basis for the reconstruction was in the real spatial domain (10 × 10 pixels in X and Y) corresponding to the FP cavity array. For the spectroscopic reconstruction, the basis vectors of the sensing matrix were the individually measured spectral transmissions of the FP cavities, while the basis for the temporal reconstruction was based on the individual time-response of the FP cavities. No additional constraints (non-negativity, etc) was imposed on the reconstruction.

## Additional Information

**How to cite this article**: Huang, E. *et al*. Ultrafast Imaging using Spectral Resonance Modulation. *Sci. Rep.* **6**, 25240; doi: 10.1038/srep25240 (2016).

## Supplementary Material

Supplementary Information
