# Supplementary material for: Ultrafast Imaging using Spectral Resonance Modulation
Source: Sci Rep. 2016 Apr 28;6:25240. doi: 10.1038/srep25240 (PMC4848519; doi:10.1038/srep25240)
Supplement: Supplementary Information [file srep25240-s1.doc]

Ultrafast Imaging using Spectral Resonance Modulation

Eric Huang, Qian Ma, and Zhaowei Liu^*^

*zhaowei@ucsd.edu

**Supplementary Figure**

**
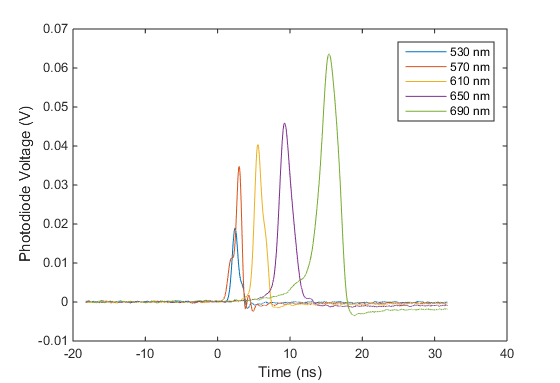
**

**Supplementary Figure 1: Pulse stretching performance of CVD**. Various wavelengths of light were isolated with a ~10nm band pass filter and sent through the CVD device, and a total of ~12 ns of pulse stretching was achieved. There is noticeable pulse broadening of the wavelengths from the CVD, which limits the bandwidth of the final measured waveform. Although wavelengths from 530 to 690 nm are shown, the bandwidth range can be easily tuned by adjusting the angle of the gratings across the range of our supercontinuum laser (500-850nm).
